# Structure–Property Relationships of PAM/CMC-Based Semi-IPN Hydrogels: Influence of APS/ANI Ratio on Swelling, Electrical, Thermal, and Morphological Properties

**DOI:** 10.1021/acsomega.6c07502

**Published:** 2026-10-15

**Authors:** Denis W. S. Nascimento, Marcia R. de Moura, Jose A. Malmonge, Fauze A. Aouada

**Affiliations:** 327017 São Paulo State University (UNESP), School of Engineering, Avenida Brasil, 56 − Centro, Ilha Solteira, São Paulo 15385-007, Brazil

## Abstract

In this work, semi-interpenetrating network (semi-IPN) hydrogels based on polyacrylamide (PAM)/CMC matrices incorporating polyaniline (PANI) were synthesized to investigate the influence of the ammonium persulfate/aniline (APS/ANI) ratio on structure–property relationships. Hydrogels were prepared via free radical polymerization of PAM/CMC followed by in situ polymerization of aniline within the preformed matrix, yielding composites with systematically varied APS/ANI ratios. Swelling studies in 1.0 mol L^–1^ HCl revealed that the incorporation of PANI reduced the equilibrium electrolyte swelling degree relative to PAM/CMC hydrogels, yet promoted specific ionic interactions with Cl^–^ ions, strongly dependent on the oxidant/monomer ratio. Electrical conductivity analysis reached values up to 10^–2^ S·cm^–1^, with the highest performance observed for hydrogels synthesized at intermediate APS/ANI ratios. Morphological (SEM/EDX) and structural (FTIR, UV–Vis, XRD) analyses demonstrated that PANI formed dendritic-like microstructures distributed within the CMC-containing matrix, while XRD results correlated crystallinity variations with electrical performance. Thermal analysis (TGA) indicated differences in the thermal degradation behavior of the composites compared with the PAM/CMC matrix. These findings highlight the central role of the APS/ANI ratio in modulating the swelling, structural organization, and electroactive properties of PAM/CMC/PANI semi-IPN hydrogels, providing insights for the design of polysaccharide-based conductive materials with potential applications in electroactive devices.

## Graphical abstract

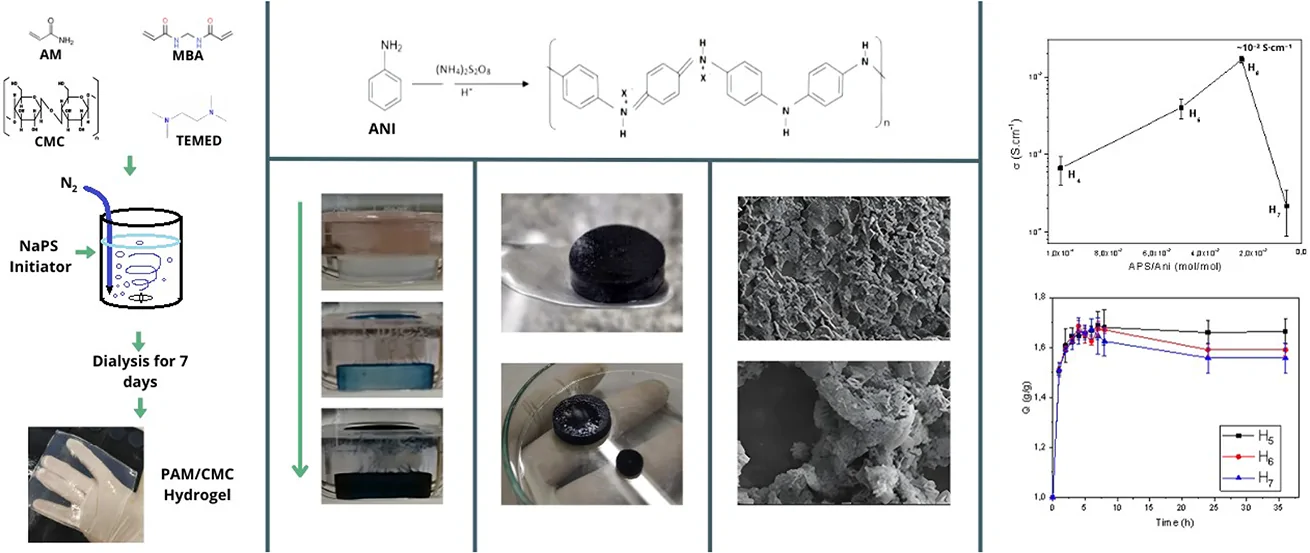

## 1 Introduction

In recent years, polysaccharide-based hydrogels have attracted considerable attention due to their sustainability, biocompatibility, and functional tunability for diverse applications including drug delivery, tissue engineering, biosensors,^1–3^ soft electronics, quasi-solid electrolyte batteries and supercapacitors.^4–7^ Among these, carboxymethylcellulose (CMC), a water-soluble anionic cellulose derivative, stands out for its excellent film-forming, swelling, and ion-exchange properties, making it a highly promising platform for functional hydrogel development.^8–11^

To improve mechanical integrity, responsiveness, and electrical functionality of CMC-based systems, including the semi-interpenetrating polymer networks (semi-IPNs)-based hydrogels have been developed by combining CMC with synthetic polymers.^12–15^ Polyacrylamide (PAM), a nonionic, hydrophilic polymer known for its high swelling capacity and mechanical resistance, has been widely used to reinforce the hydrogel matrix and provide structural integrity in various composite systems.^16–19^ The blending of CMC with PAM creates a synergic matrix, where hydrogen bonding and entanglement between chains improve not only the swelling kinetics but also enable the incorporation of functional dopants.^20–22^

In particular, the integration of polyaniline (PANI) into polysaccharide-based semi-IPNs has emerged as an efficient strategy to introduce electronic conductivity into the hydrophilic network.^23–25^ PANI is a pH-responsive conductive polymer that can be synthesized in situ within the hydrogel network through oxidative polymerization of aniline (ANI), yielding materials that exhibit both ionic and electronic conductivity, alongside flexibility and environmental stability.^26–28^ However, the structure–property relationships of PAM/CMC/PANI composites remain insufficiently understood, particularly with regard to the oxidant-to-monomer ratio (APS/ANI), which can affect PANI formation, its chemical state and distribution within the matrix, and its interactions with the host polymer network.^29–31^ Recent studies on PANI-based hybrid materials have further demonstrated that changes in the chemical environment and structural organization of PANI can significantly affect its optical, thermal, and electrical properties these matrices.^32,33^ Previous studies have demonstrated the feasibility of combining CMC, PAM, and PANI to obtain conductive interpenetrating hydrogel networks for electroactive applications. However, the influence of the APS/ANI molar ratio on the resulting structure–property relationships of PAM/CMC/PANI semi-IPN hydrogels remains insufficiently addressed.

In this context, we report the synthesis and detailed physicochemical characterization of PAM/CMC/PANI semi-IPN hydrogels, with particular emphasis on the effect of the APS/ANI molar ratio. Rather than focusing only on the incorporation of PANI into the hydrogel matrix, this study systematically examines how changes in the oxidant-to-monomer ratio affect swelling, electrical conductivity, chemical structure, morphology, and structural organization. By combining these complementary characterizations, the work aims to clarify the structure–property relationships governing the electrical response of the composite hydrogels and to identify a favorable synthesis window for the development of conductive polysaccharide-based materials. This study provides insights into the complementary roles of the natural and synthetic polymeric components in conductive hydrogel matrices and offers design principles for synthesizing functional carbohydrate-based materials for electroactive applications.

## 2 Experimental Section

### 2.1 Materials

Carboxymethylcellulose (CMC, Mw = 114 × 10^3^ g·mol^–1^), sodium persulfate (NaPS, Na_2_S_2_O_8_), and hydrochloric acid (HCl, 37%) were obtained from Synth (Brazil). Acrylamide monomer (AM, C_3_H_5_NO) and the cross-linker N,N′-methylenebis­(acrylamide) (MBAM, C_7_H_10_N_2_O_2_) were purchased from Vetec Química (Brazil). The catalyst N,N,N′,N′-tetramethylethylenediamine (TEMED, C_6_H_16_N_2_), aniline monomer (Ani, C_6_H_7_N), and ammonium persulfate (APS, (NH_4_)_2_S_2_O_8_) were supplied by Sigma-Aldrich (US). All chemicals were analytical grade and used without further purification. Deionized water was used in all experimental procedures.

### 2.2 Preparation of PAM/CMC Hydrogels

First, PAM/CMC hydrogels were obtained through free radical polymerization by solubilization of 6.0% (w/v) of AM monomer and 1.0% (w/v) of CMC into 27 mL of distilled water under magnetic stirring. Afterward, 0.3% (w/v) of methylenebis­(acrylamide) (MBA) cross-linking agent was added. Then, a 0.2 mol L^–1^ solution of the catalyst N,N,N′,N′,-tetramethylenediamine (TEMED) was also added. The resulting solution was subjected to a nitrogen gas (N_2_) atmosphere for 20 min to remove oxygen. Finally, 2.0 mL of 1.25% (w/v) of sodium persulfate (NaPS) aqueous solution was used to start the reaction. The resulting solution was kept in a mold at room temperature for 24 h. After polymerization, the obtained hydrogels were dialyzed in distilled water for 7 days. The dialyzed water was renewed every 24 h to remove the reactants that did not participate in the reaction.

### 2.3 PAM/CMC/PANI Composite Synthesis

The PAM/CMC/PANI composite hydrogels were obtained by in situ polymerization of ANI in PAM/CMC hydrogels, which were also used as a reference and denoted as H_0_. The H_0_ hydrogels were dried and cut into circular shapes. The hydrogels were first swollen in an HCl solution (1.0 mol L^–1^) containing ANI monomer (Table 1). Subsequently, the ANI-loaded hydrogels were transferred to beakers containing 10 mL of an HCl solution (1.0 mol L^–1^) with 5.5% (w/v) APS and kept under magnetic stirring at room temperature for 24 h. Subsequently, all PAM/CMC/PANI composites were dialyzed in HCl solution (1.0 mol L^–1^) for 3 days, where the HCl solution was changed every 24 h. This procedure was adopted to remove soluble unreacted species while maintaining the acidic environment required to favor the protonated state of PANI. The HCl dialysis treatment was applied under the same conditions to all PANI-containing hydrogels (H_1_–H_7_).

**Table 1:** Formulations of PAM/CMC/PANI Semi-IPN Hydrogels Prepared in Two Sequential Experimental Stages at Different APS/ANI Molar Ratio

Hydrogel	ANI (%w/v)	APS/ANI (mol/mol)
H_0_	-	-
H_1_	0.6	1.2
H_2_	0.7	1.0
H_3_	1.5	5.0 × 10^–1^
H_4_	7.5	1.0 × 10^–1^
H_5_	7.5	5.0 × 10^–2^
H_6_	7.5	2.5 × 10^–2^
H_7_	7.5	6.25 × 10^–3^

The selection of the APS/ANI ratio was performed in two sequential experimental stages. The initial APS/ANI ratio of 1.2 (mol/mol) was selected based on the favorable initiator/aniline ratio for a PANI/PAM conducting hydrogel, reported by Tang et al.^34^ In the first stage, the APS concentration was kept constant while the ANI concentration was progressively increased, resulting in APS/ANI ratio of 1.2, 1.0, 0.5, and 0.1 (mol/mol), corresponding to H_1_, H_2_, H_3_, and H_4_, respectively. After evaluation of the swelling behavior and four-point electrical conductivity, H_4_ was selected as the reference composition for the second stage because it exhibited measurable electrical conductivity. In the second stage, the ANI concentration was maintained at the H_4_ level, while the APS concentration was progressively reduced to investigate the lower-oxidant region. Thus, APS/ANI ratio of 0.05, 0.025, and 0.00625 (mol/mol) were used for H_5_, H_6_, and H_7_, respectively. The APS and ANI concentrations used in the hydrogel syntheses are summarized in Table 1.

### 2.4 Swelling Degree (Q) in Electrolyte

After purification, the H_0_ hydrogel and PAM/CMC/PANI composites were dried in an oven at 40 °C until constant weight was achieved. The electrolyte absorption capacity was investigated in triplicate by measuring the swelling degree (Q) in HCl (1.0 mol L^–1^), The swelling degree was calculated as the ratio between the mass of the swollen hydrogel (M_t_) and its dried mass (M_0_), according to (eq 1), and is reported in g/g.

1 $$Q=\frac{M_{t}}{M_{0}}$$

### 2.5 Electrical Conductivity

Electrical conductivity was determined using the standard four-probe method. A Keithley programmable source meter (Model 236) and an HP 34401A digital voltmeter were employed. The electrical conductivity (*σ*) was calculated according to (eq 2), following the ASTM F43-99 standard:

2 $$\sigma =\frac{0.22.I}{d.V}$$

where 0.22 is the correction factor that accounts for the relationship between the probe spacing (s) and the sample thickness dimension (d) when d ≅ s; (I) is the applied current and (V) is the measured potential difference.

### 2.6 Fourier-Transform Infrared Spectroscopy (FTIR)

Fourier transform infrared (FTIR) spectra were acquired using a Nicolet Nexus 670 FT-IR spectrophotometer. Prior to analysis, oven-dried samples were ground, mixed with potassium bromide (KBr) at a ratio of 200:1 (KBr/sample), and pressed into pellets. Measurements were performed in transmittance mode over the range of 4000–400 cm^–1^, with 128 scans collected at a resolution of 4 cm^–1^.

### 2.7 Ultraviolet–Visible Spectroscopy (UV–Vis)

UV–Vis spectra of PANI were recorded using a Shimadzu UV-2700 spectrophotometer over the wavelength range of 190–900 nm. Measurements were carried out using 1.0 mol L^–1^ HCl as the blank in a quartz cuvette. Immediately after the synthesis, aliquots of the reaction mixture from PAM/CMC/PANI hydrogels (H_5_, H_6_, and H_7_) were collected and transferred to a second quartz cuvette for analysis.

### 2.8 Morphological Analysis

Morphological analysis was performed using a ZEISS EVO SEM (model LS15) at an accelerating voltage of 10 kV. Micrographs were obtained for PAM/CMC/PANI hydrogels (H_5_, H_6_, and H_7_) and for the control hydrogel (H_0_). Samples were swollen in 1.0 mol L^–1^ HCl, cut into approximately 1 cm^3^ pieces, frozen in liquid nitrogen, and lyophilized for 24 h. All samples were cryo-fractured to expose their internal structure. The fractured surfaces were coated with an approximately 15 nm thick gold layer using a BALZERS SDC 50 sputter coater to enable SEM analysis. The average pore size and pore size distribution were estimated from SEM micrographs using ImageJ software (National Institutes of Health, USA). For H_0_, multiple regions of the micrograph were analyzed, and approximately 20 identifiable pores were randomly selected to determine the mean pore diameter and standard deviation. For the PANI-containing hydrogels, the morphological features did not allow reliable identification and segmentation of individual pores. Therefore, the quantitative pore-size measurements were not assigned to H_5_–H_7_.

### 2.9 Chemical Composition

The chemical composition of the hydrogel and PANI-based composites was analyzed using energy-dispersive X-ray spectroscopy (EDX). An Oxford Instruments energy-dispersive X-ray spectrometer (INCA X-act model) with a resolution of 100 eV was used for this purpose. The elemental composition was expressed as normalized weight percentages (wt %) of the detected elements.

### 2.10 Structural Morphology

The structural morphology, PANI arrangement within the hydrogels, and sample crystallinity were investigated by X-ray diffraction (XRD) using a Shimadzu XRD-6000 diffractometer. The measurements were performed with Cu Kα radiation (λ = 0.1541 nm), operating at 30 kV and 40 mA, over a 2θ range of 5–60°, with a scan rate of 1° min^–1^. Prior to analysis, the samples were dried, ground into powder, and placed in glass sample holders.

The average degree of crystallinity X_c_ was determined according to (eq 3):

3 $$X_{C}\left(\%\right)=\frac{Crystalline\;area\times 100}{Total\;area}$$

### 2.11 Thermogravimetric Analysis (TGA)

The thermal properties of the samples were investigated by thermogravimetry (TG). Approximately 7–10 mg of each sample was placed in a platinum pan and analyzed using a TA Instruments SDT Q600 thermal analyzer. Measurements were carried out under a nitrogen atmosphere with a flow rate of 100 mL min^–1^ for both the balance and the sample compartments. The samples were heated from room temperature to 800 °C at a constant heating rate of 10 °C min^–1^.

## 3 Results and Discussion

### 3.1 Swelling Degree (Q)

Figure 1 shows the dependence of swelling degree (Q) values as a function of time for different hydrogels. The equilibrium swelling degree (Qeq) was reached in approximately 36 h. Incorporation of PANI into the hydrogel matrix initially decreased swelling compared to the control hydrogel H_0_. However, increasing the PANI content from H_1_ to H_3_ led to progressively higher swelling, with the equilibrium swelling degree reaching 11.3 ± 0.8 g/g for H_4_. This suggests PANI increases physical cross-linking within the PAM/CMC network, leading to a more rigid 3D structure that restricts solvent uptake. Conversely, higher PANI content also introduces NH^+^ and NH_4_^+^ groups, enabling electrostatic interactions with Cl^–^ ions, which enhance electrolyte diffusion into the polymer matrix and contribute to increased swelling up to H_4_. Based on these results, H_4_ was therefore selected for further studies involving variation of the APS concentration.

**Figure 1:**
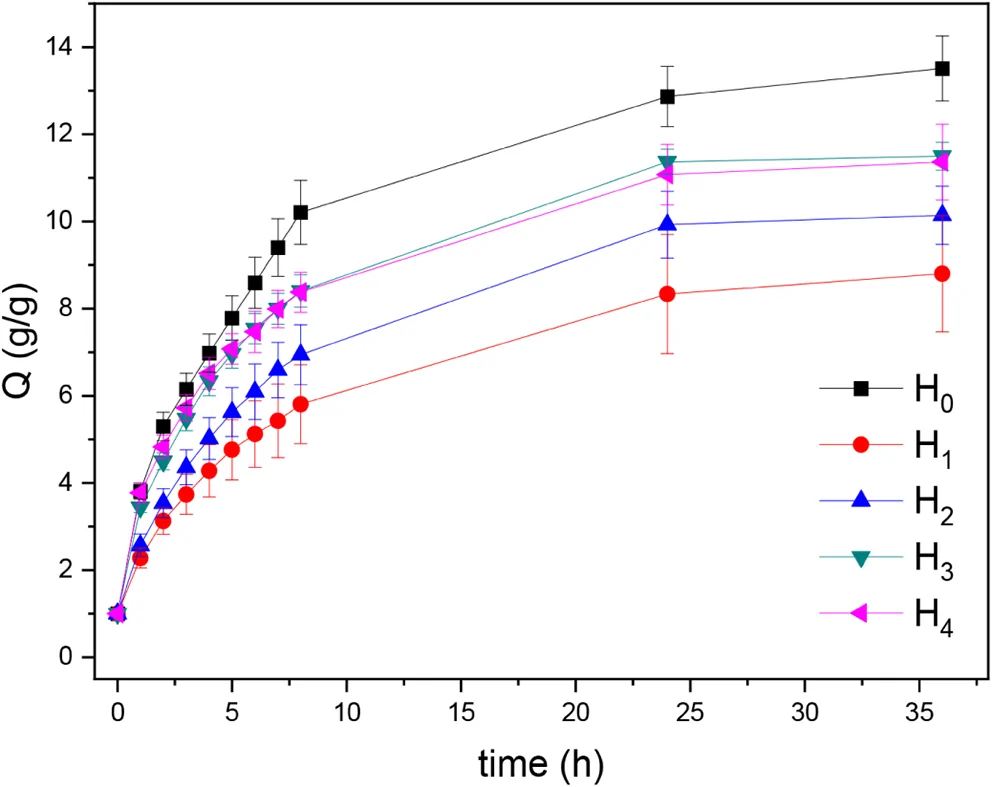
Swelling degree (Q) of PAM/CMC and PAM/CMC/PANI hydrogels (H_0_–H_4_) in 1 mol·L^–1^ HCl as a function of time.

Hydrogels H_5_, H_6_, and H_7_ were also examined for HCl uptake, with equilibrium reached within approximately 5 h, indicating faster swelling process than those observed for H_0_–H_4_ (Figure 2). The equilibrium swelling degree was substantially lower for H_5_–H_7_, with equilibrium values ranging from 1.55 to 1.70 g/g. A clear dependence on the APS/ANI ratio was also observed. For instance, H_5_ prepared with the highest APS concentration within this series, exhibited the highest equilibrium swelling. Already hydrogel H_7_, prepared with the lowest APS concentration, exhibited the lowest value. Because the ANI concentration was kept constant for H_5_–H_7_, this variation in swelling coincided with the progressive reduction in APS concentration. The lower swelling capacity of H_5_–H_7_ compared with H_0_–H_4_ may be associated with changes in PANI formation and network organization resulting from the different APS/ANI ratios, which can restrict electrolyte uptake.

**Figure 2:**
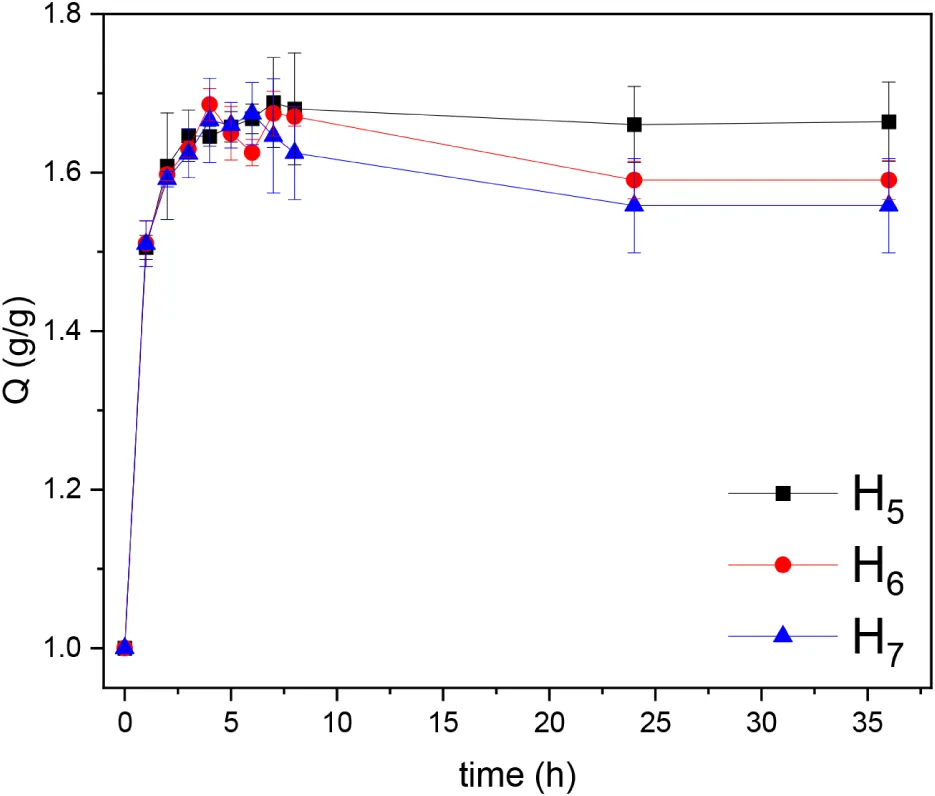
Swelling degree (Q) of PAM/CMC/PANI hydrogels (H_5_–H_7_) in 1 mol·L^–1^ HCl as a function of time.

### 3.2 Electrical Conductivity

Figure 3 presents the electrical conductivity of samples H_4_–H_7_. The remaining samples (H_0_–H_3_) exhibited conductivity values below the detection limit, indicating insufficient formation of conductive pathways under these synthesis conditions. In contrast, hydrogel H_4_ displayed measurable conductivity and was therefore selected as the reference for subsequent studies involving variation of the APS concentration.

**Figure 3:**
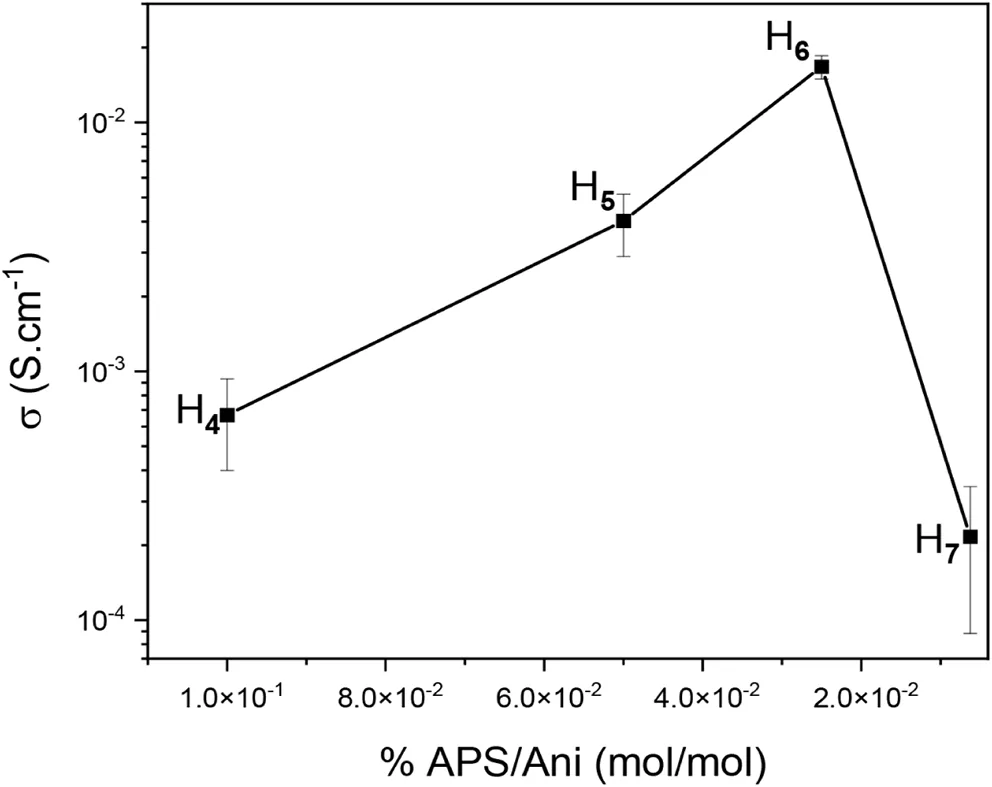
Electrical conductivity (σ) of PAM/CMC/PANI hydrogels (H_4_–H_7_) as a function of APS/ANI ratio.

The electrical conductivity of hydrogels H_4_–H_7_ increases with decreasing APS/ANI molar ratio, reaching a maximum of approximately 10^–2^ S cm^–1^ for H_6_. However, a further decrease in APS concentration (H_7_) leads to a sharp drop in conductivity of nearly 2 orders of magnitude. This nonmonotonic behavior indicates that an intermediate APS/ANI ratio provides the most favorable conditions for electrical conduction under the synthesis conditions investigated. In contrast, the further reduction of the APS/ANI ratio in H_7_ resulted in a pronounced decrease in conductivity. Because the ANI concentration was maintained at 7.5% (w/v) for H_5_–H_7_, this decrease is associated with the substantially lower APS availability and may reflect reduced oxidative polymerization of aniline and less effective formation of interconnected conductive domains. Conversely, the higher APS/ANI ratio used for H_5_ did not improve conductivity relative to H_6_. Although a high oxidant concentration may affect the chemical structure of PANI, the present results do not provide sufficient evidence to conclusively attribute the H_5_ behavior to overoxidation.

The conductivity trend can be further interpreted in association with the structural and spectroscopic characterizations. H_6_ exhibited a more pronounced polaron-related FTIR band, together with comparatively more defined UV–Vis absorption features associated with the doped PANI state. In addition, SEM revealed a more homogeneous PANI distribution, while XRD indicated a higher calculated crystalline fraction for H_6_. The convergence of these results with the conductivity maximum suggests that the electrical response is influenced not only by the amount of oxidant used during polymerization, but also by the resulting chemical state, structural organization, and morphology of the PANI phase within the hydrogel network.

The electrical conductivity obtained in the present study is comparable to the values reported for other CMC/PANI-based conductive hydrogels, being within the same order of magnitude. However, a direct comparison should be made cautiously because the hydrogel composition, PANI content, doping conditions, and conductivity measurement methods differ among studies. For instance, Suganya et al.^45^ reported conducting CMC/PAM/PANI hydrogels prepared through a two-step interpenetrating-network polymerization involving in situ polymerization of aniline, demonstrating the potential of this type of architecture for electroactive applications. Similarly, Li et al.^46^ reported that the semi-IPN CMC/PANI hydrogels had conductivity values up to 6.31 × 10^–3^ S cm^–1^ after sodium benzenesulfonate doping. More recently, CMC-assisted PANI has also been incorporated into PAM-based double-network hydrogels for flexible electroactive devices.^47^ In comparison with these studies, the present work focuses specifically on the effect of the APS/ANI molar ratio. The systematic variation of this parameter revealed a nonmonotonic conductivity response. The combined conductivity, spectroscopic, morphological, and structural results provide additional insight into how the oxidant-to-monomer ratio influences the organization of the conductive PANI phase within the hydrogel network.

### 3.3 Spectroscopic Analysis (FTIR/UV–Vis)

The FTIR spectra obtained, shown in Figure 4, provide important insights into the molecular interactions and confirm the successful incorporation of PANI into the hydrogel network.

**Figure 4:**
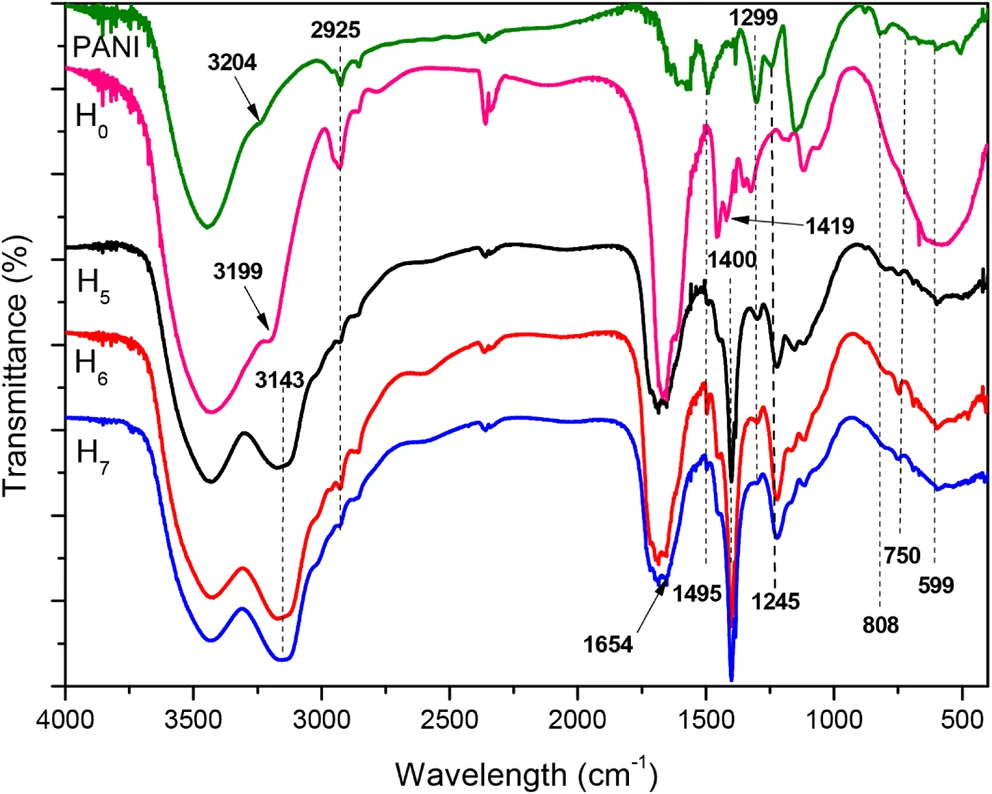
FTIR spectra of HCl-doped PANI, control PAM/CMC hydrogel (H_0_), and PAM/CMC/PANI composite hydrogels (H_5_–H_7_).

The spectrum of emeraldine salt PANI displays characteristic bands confirming its formation in the conductive state. A broad band near 3204 cm^–1^ is associated with the N–H stretching of aromatic amines, while the band at 1299 cm^–1^ corresponds to N–H stretching of secondary aromatic amines. Another significant feature is the band at 1245 cm^–1^, attributed to the C–N^+^=C stretching of polaron structures, which is a key indicator of the conductive form of PANI. The peaks around 1600 cm^–1^ corresponds to N–H bending vibration of PAM and exhibits a red shift, related to hydrogen bonding interactions between PAM and PANI. Additionally, bands at 808, 750, and 599 cm^–1^, associated with out-of-plane C–H bending and ring deformations, are typical of PANI’s aromatic structure and further confirm its chemical identity.^38–40^

In the spectrum of the H_0_, bands at 3431 and 3199 cm^–1^ arise from symmetric and asymmetric N–H_2_ stretching, while bands at 2925 and 2860 cm^–1^ reflect CH stretching vibrations, visible in all materials too. Signals at 1667 and 1419 cm^–1^ correspond to C=O and C–N deformations from amide bonds within PAM and MBAM cross-links. The band at 1617 cm^–1^ is assigned to N–H bending vibration, and the signal at 1189 cm^–1^ is characteristic of β-glycosidic C–O–C stretching, indicative of the CMC structure. Bands at 666 cm^–1^ are also present, corresponding to out-of-plane N–H bending vibrations.^38–42^

For the PAM/CMC/PANI hydrogels H_5_, H_6_ and H_7_, significant spectral changes are observed, indicating effective incorporation of PANI into the hydrogel matrix. The band near 3204 cm^–1^ shifts slightly and intensifies to 3143 cm^–1^, suggesting stronger hydrogen bonding interactions between the amine groups of PANI and the hydrogel matrix. The characteristic bands of the hydrogel backbone, such as the C=O deformation at 1653 cm^–1^ and N–H bending at 1601 cm^–1^, remain visible, demonstrating that the hydrogel’s structure is preserved even after PANI integration.

The presence of the band at 1299 cm^–1^ in the composite hydrogels is consistent with the retention of N–H stretching vibrations from secondary aromatic amines, verifying PANI’s incorporation. An evident shift and intensification of the band around 1419 cm^–1^, attributed to C=C stretching, is also noted due to vibrations of the benzenoid rings of PANI. Most notably, the band at 1245 cm^–1^, assigned to the C–N^+^=C stretching of polarons, becomes markedly more intense across the composite hydrogel spectra, especially in hydrogel H_6_. This spectral feature directly correlates with polaron formation, which is crucial for the material’s conductive properties.

These spectral variations support the incorporation of PANI into the PAM/CMC hydrogel network. The changes suggest interactions, particularly hydrogen bonding, between PANI’s amine groups. This interaction likely contributes to enhanced mechanical stability and facilitates the formation of conductive pathways within the hydrogel matrix.

Notably, the higher intensity of the 1245 cm^–1^ band observed in hydrogel H_6_ correlates well with its higher electrical conductivity measured in prior analyses. This result suggests that the intensity of this polaron-related band can serve as a spectroscopic indicator of conductive network formation. Hydrogels H_5_ and H_7_ show comparatively lower intensity at this band, aligning with their lower measured conductivities. Overall, these FTIR findings confirm that the optimization of the APS/ANI ratio is crucial for achieving the best electrical performance, with hydrogel H_6_ exhibiting the most favorable balance between structural integrity and conductivity.

Thus, FTIR spectroscopy not only validates the successful incorporation of PANI into the hydrogel matrix but also offers a valuable tool for correlating molecular structure with electrical properties, supporting the selection of optimal synthesis conditions for conductive hydrogel composites.

UV–vis spectroscopy was employed to investigate the electronic transitions and doping state of PANI synthesized in the PAM/CMC hydrogel matrix. The electronic structure of conjugated polymers such as PANI is highly sensitive to their oxidation state and dopant levels, both of which can be inferred from specific absorption features in the UV–vis region. The absorption spectra of PANI extracted from hydrogels H_5_, H_6_, and H_7_. Figure 5 exhibits three main bands. The first, located around 290 nm, is attributed to the π–π* transition of benzenoid rings, characteristic of the conjugated backbone of PANI. The second band, near 420 nm, is associated with the polaron−π* transition, indicating the presence of delocalized polarons and confirming that the polymer is in its doped emeraldine salt form.^38,41^ The third one, broader band Around 780–820 nm, is attributed to polaron/bipolaron transitions arising from protonation of imine sites, further supporting the formation of conductive polaronic structures within the PANI chains.^38,41^

**Figure 5:**
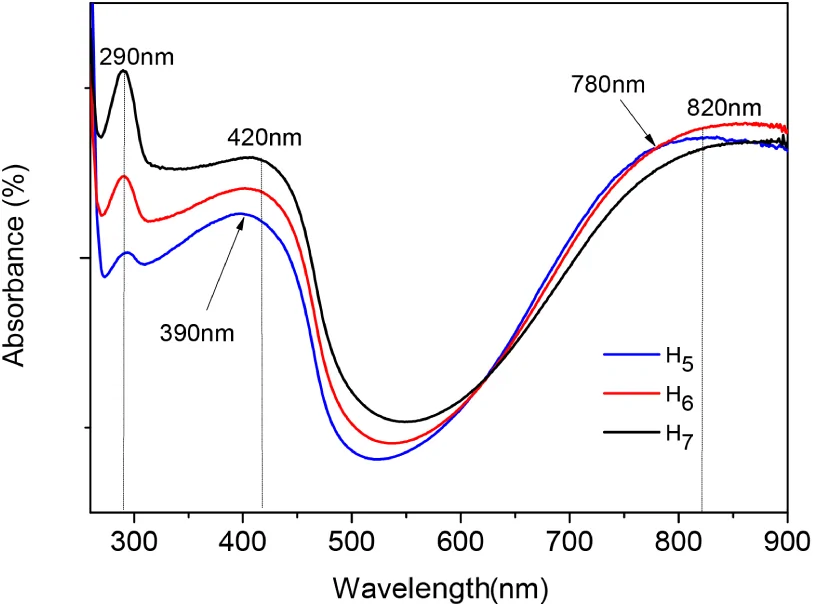
UV–Vis absorption spectra of PAM/CMC/PANI hydrogels.

Comparative analysis reveals clear differences as a function of the APS/ANI molar ratio. For hydrogel H_5_, the π–π* transition of benzenoid rings at 290 nm remains relatively intense, indicating preserved conjugation. However, the bands near 420 nm, relating to polaron-π* transitions of quinonoid rings, and 820 nm, relating to π-polaron transitions, are both blue-shifted and reduced in intensity, suggesting lower polaron density and a reduced doping level.^35–37^ These results are consistent with the lower electrical conductivity observed, as reported previously. In contrast, hydrogel H_6_ displays well-defined and intensified bands at 420 and 820 nm, indicating a higher degree of doping and a more pronounced formation of conductive polaronic species. These features confirm that H_6_ exhibits the most favorable electronic structure among the samples, correlating directly with its maximum electrical conductivity. For H_7_, the overall decrease in absorption intensity, while retaining the characteristic features of doped PANI, indicates a lower contribution of polaron-related species to the measured spectrum. Considering that H_7_ was synthesized with the lowest APS/ANI ratio, this behavior may be associated with reduced oxidative polymerization of aniline and/or a lower amount of PANI formed within the hydrogel matrix.

These results reveal a consistent relationship between the UV–Vis spectral features and the electrical behavior of the hydrogels. The intensity and position of the polaron-related absorption bands can be reliably used to assess the efficiency of the doping process and the conductive state of PANI in hydrogel matrices. The data also confirm that the APS/ANI ratio could be a key parameter controlling the formation of conductive domains, with H_6_ representing the most favorable composition among the formulations investigated for achieving enhanced electronic performance.

### 3.4 Morphological Characterization (SEM/EDX)

Figure 6 shows SEM micrographs of the surfaces of the H_5_, H_6_, and H_7_ composites. In H_5_, although PANI is present, its distribution is less homogeneous and includes visible surface cracks and irregular aggregates. For H_6_, the surface appears more uniform and continuous, with fewer defects compared to H_5_ and H_7_ hydrogels, which may favor charge transport along the surface, contributing to its superior conductivity. For H_7_, the surface appears smoother and less structured, with less pronounced PANI features than those observed for H_5_ and H_6_. This observation is consistent with a lower degree of PANI formation and/or less effective organization of the conductive phase, which may contribute to the lower conductivity.

**Figure 6:**
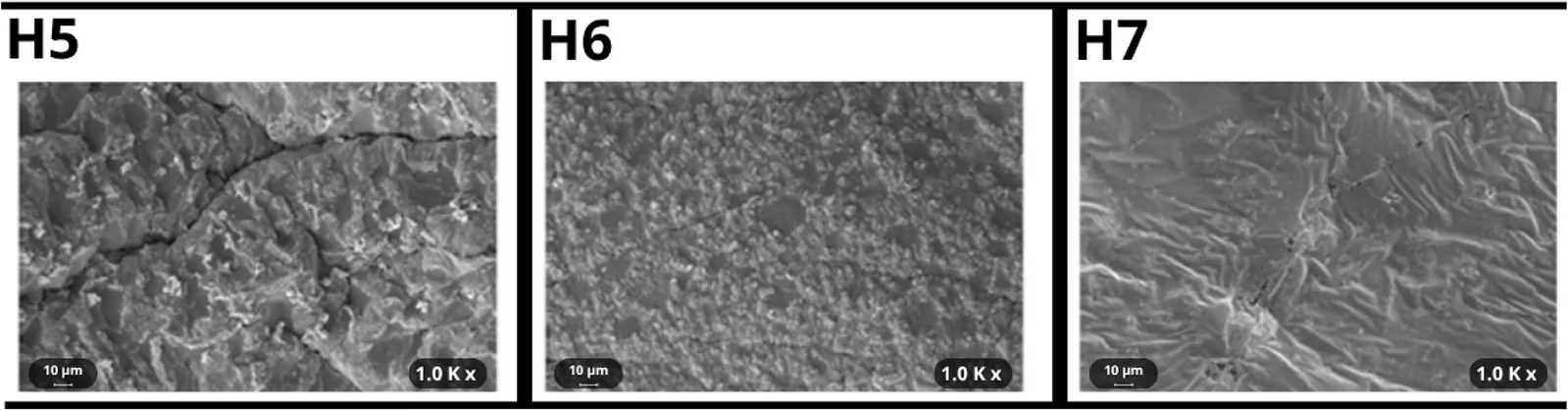
SEM micrographs of PAM/CMC/PANI composites at 1000× magnification, showing the surface structure of the progressive incorporation and distribution of PANI.

Cryogenic fracture analysis, presented in Figure 7, provides a view of the internal distribution of PANI within the composite matrix. While H_0_ exhibits clearly identifiable heterogeneous pore shapes and foliaceous layers, individual pore boundaries could not be reliably distinguished in the cross-sectional images of H_5_, H_6_, and H_7_. Instead, these samples exhibited denser and more compact internal morphologies, with PANI aggregates occupying and/or covering regions of the original porous network. This morphological change is consistent with the substantially lower swelling capacity observed for the PANI-containing hydrogels.

**Figure 7:**
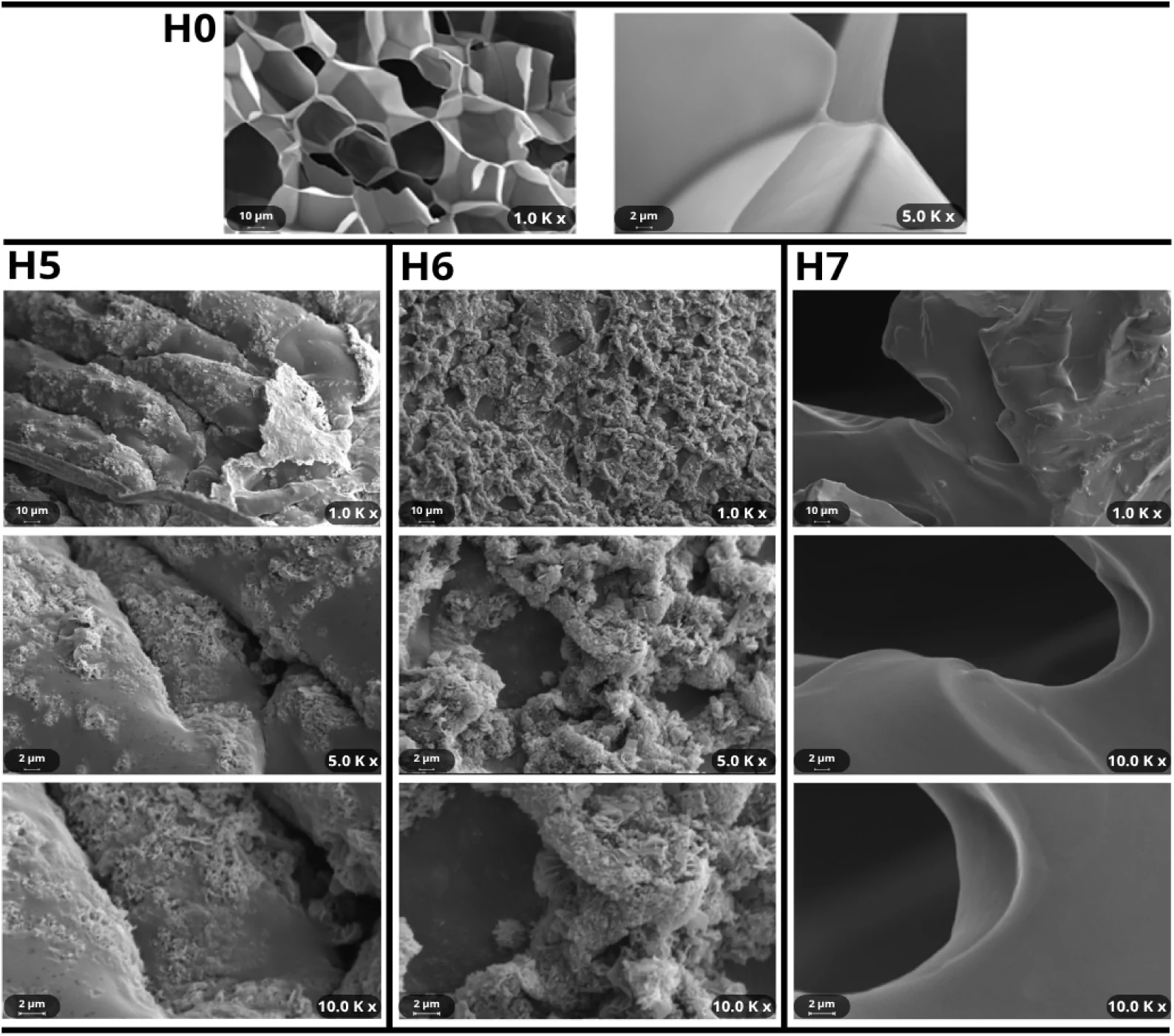
SEM micrographs of cryogenic fractured samples of control and PAM/CMC/PANI hydrogels at different magnifications.

The layered features of H_0_ are consistent with the typical morphology of polysaccharide-based hydrogels. The average pore size was estimated at 60 ± 20 μm, based on the analysis of 20 pore regions, confirming a well-developed 3D porous network favorable for swelling and fluid retention.

In contrast, the incorporation of PANI into the hydrogel matrix resulted in a significant morphological transformation. In all composite samples, PANI appeared as aggregated clusters within and over the porous regions. These aggregates tend to partially or fully fill the pores, altering the overall texture and reducing apparent porosity. Furthermore, the apparent size and density of PANI aggregates varied with the APS/ANI ratio, suggesting that the APS/ANI ratio influenced the extent of PANI formation.

Qualitatively, the SEM micrographs suggest that decreasing the APS/ANI ratio was accompanied by smaller and less prominent PANI aggregates and reduced surface coverage. This suggests a reduced formation of PANI with lower oxidant concentrations, consistent with the electrical conductivity data and the UV–vis analysis.

Among the composite hydrogels, H_6_ shows again the most homogeneous internal distribution of PANI, with dendritic-like microstructures and fewer voids. The presence of dendritic or fractal-like crystals in H_6_, at 5000× and 10000× magnification, reflects a controlled and organized growth mechanism of PANI. These dendritic-like patterns, known for their self-similar or fractal geometry, support the formation of highly interconnected conducting domains. The uniformity and distribution of sub-5 μm dendritic crystallites in H_6_ highlight the efficiency of the synthesis protocol and align with its enhanced electrical conductivity.

The SEM analysis thus reinforces the structure–property relationships discussed throughout this study, where the optimal APS/ANI ratio in H_6_ not only promotes efficient PANI synthesis but also leads to uniform surface coating, favorable internal distribution, and morphologies conducive to electrical conduction. These observations indicate that morphological changes associated with the APS/ANI ratio may contribute to the tuning of the functional properties of PAM/CMC/PANI composite hydrogels.

To complement the morphological characterization, EDX analysis was employed to determine the elemental composition of H_5_–H_7_. Figure 8 presents an example of representative EDX, obtained for hydrogel H_6_, showing the characteristic signals of C, N, O, and Cl elements. Elemental compositions in the three composite hydrogels are summarized in Table 2.

**Figure 8:**
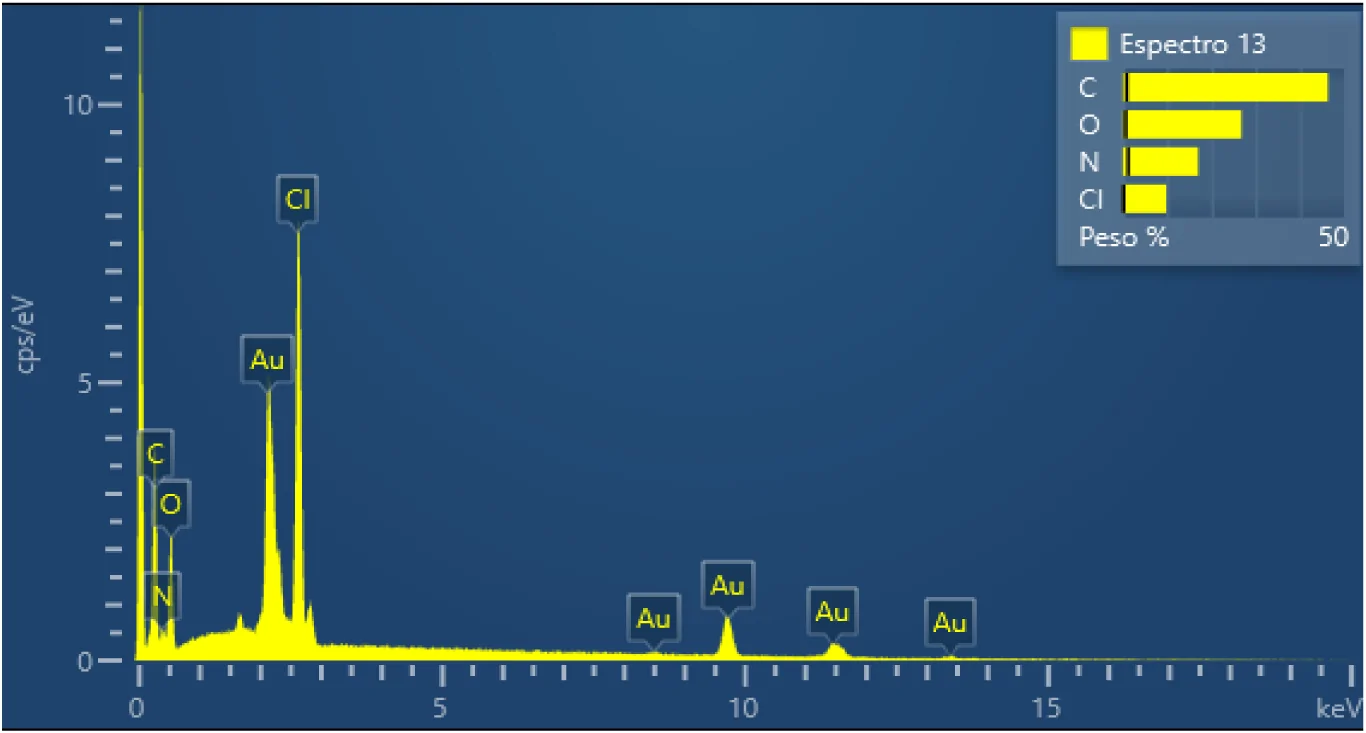
EDX spectrum of H_6_ hydrogel.

**Table 2:** Elemental Composition of PAM/CMC/PANI Hydrogels H_5_, H_6_, and H_7_ Obtained by EDX Analysis

Hydrogel	C (wt %)	N (wt %)	O (wt %)	Cl (wt %)
H_5_	44.64	15.26	30.17	9.93
H_6_	46.35	17.01	26.81	9.83
H_7_	44.58	16.36	27.93	11.13

H_6_ therefore exhibited the highest N percentage among the investigated samples, which is consistent with a greater contribution of nitrogen-containing PANI to the composite. However, because PAM also contains nitrogen, the nitrogen signal cannot be used independently to quantify PANI incorporation. The highest Cl content was observed for H_7_, despite its substantially lower electrical conductivity than H_6_. For that reason, the total Cl content does not show a direct correlation with conductivity and should not be considered an independent measure of the PANI doping level. The detection of Cl in the composite hydrogels is consistent with the acidic treatment and the presence of chloride associated with the protonated polymer system. These results indicate that the electrical performance depends not only on the elemental composition but also on the structural organization and electronic state of the PANI phase.

### 3.5 Structural Analysis (XRD)

XRD patterns (Figure 9) show broad halos characteristic of predominantly amorphous materials, with a broad diffraction halo between 15° < 2θ < 35°, typical of cross-linked polymeric hydrogels. Although minor diffraction features are observed, their low intensity and poor definition prevent reliable crystallite size determination. The results indicate that PANI incorporation does not significantly alter the amorphous nature of the PAM/CMC matrix but may introduce localized ordering associated with PANI domains. The greater structural organization observed for H6 is consistent with its higher conductivity and comparatively more homogeneous morphology.

**Figure 9:**
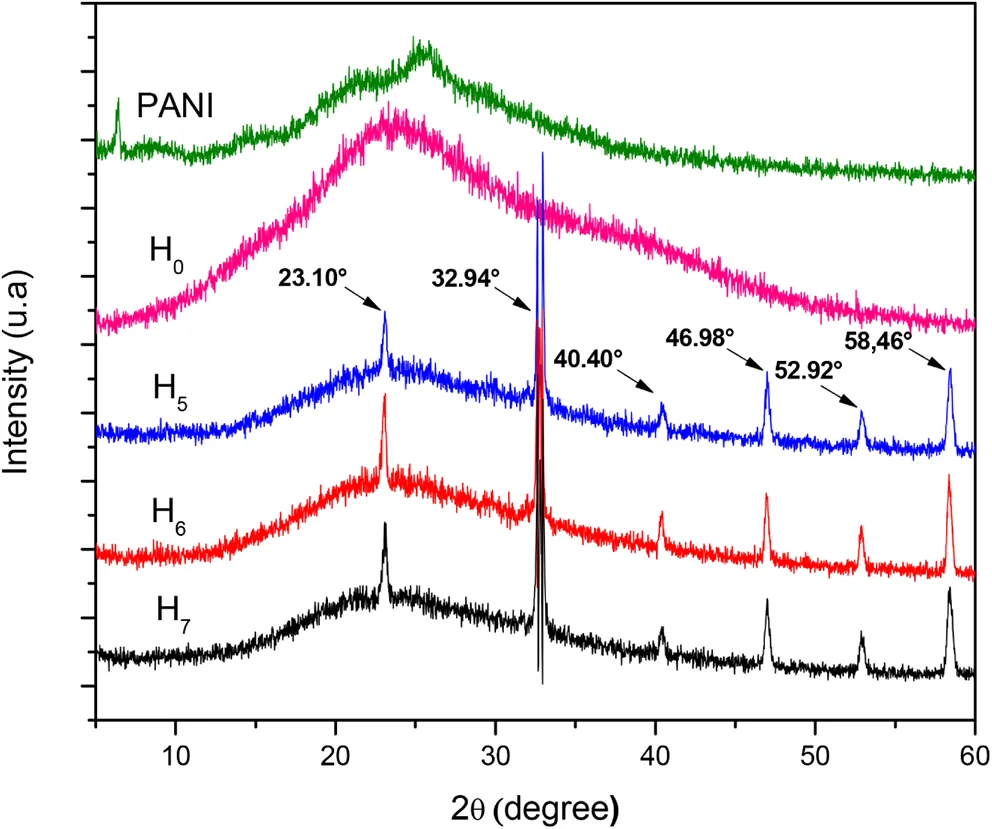
X-ray diffraction patterns of pure PANI, the control hydrogel, and PAM/CMC/PANI composite hydrogels.

However, several minor diffraction peaks emerge at 2θ = 23.10°, 32.94°, 40.40°, 46.98°, 52.92°, and 58.46°, which could be attributed to the formation of microcrystalline regions.^42–44^ These peaks are correlated with the dendritic and fractal-like growth of PANI structures observed by SEM, further substantiating the presence of ordered PANI aggregates embedded within the hydrogel matrix.

Crystalline fractions were determined using the deconvolution method, comparing the area under the crystalline peaks to the total diffracted area. The results indicate a crystallinity of 21% for H_6_, 17% for H_5_, and 7% for H_7_. The highest degree of crystallinity observed in H_6_ corroborates its enhanced electrical conductivity and uniform PANI distribution, as previously discussed. In summary, XRD results support the idea that PANI incorporation introduces semicrystalline domains into the amorphous hydrogel matrix, with the degree of ordering being highly sensitive to the APS/ANI ratio. The observed trend in crystallinity (H_6_ > H_5_ > H_7_) reflects the hierarchy established through conductivity and spectroscopic analyses, reinforcing the structure–property relationship in these conductive hydrogel composites.

### 3.6 Thermal Stability

Thermogravimetric (TG) and differential thermogravimetric (DTG) analyses were employed to evaluate the thermal stability and degradation behavior of the materials, Figure 10.

**Figure 10:**
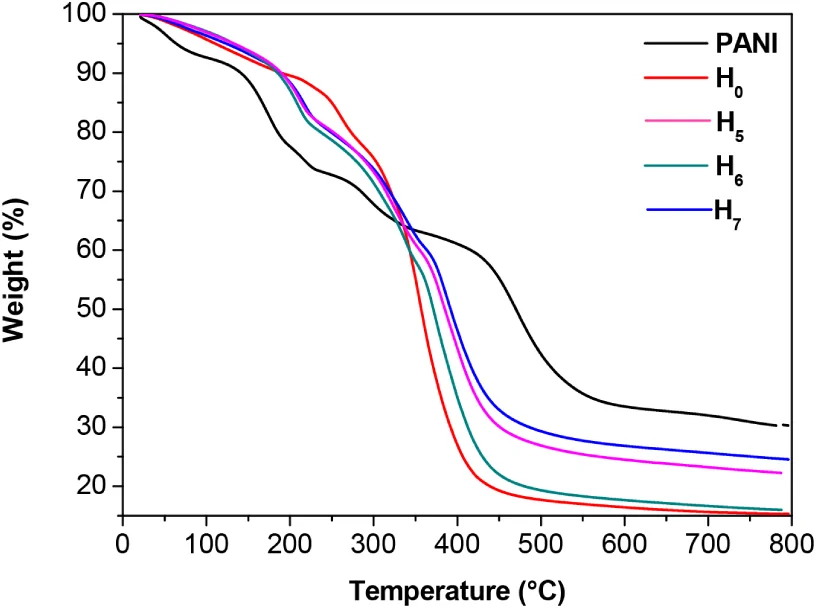
Thermogravimetric (TG) curves of PANI, control hydrogel, and PAM/CMC/PANI composites H_5_, H_6_, H_7_.

The TG profiles reveal distinct degradation steps for PANI, H_0_, and the PAM/CMC/PANI composites. For the composites H_5_, H_6_ and H_7_, four major events were observed: (i) weight loss below 110 °C due to water evaporation; (ii) T = 110–250 °C, associated with removal of residual dopant and low-molecular fragments, with an average loss of ∼16.7%, lower than neat PANI; (iii) T = 249–365 °C, with higher weight loss compared to H_0_ or PANI alone, suggesting overlapping degradation of both PAM/CMC matrix and PANI chains; and (iv) T = 365–617 °C, where composites displayed higher onset and final degradation temperatures than H_0_, confirming that incorporation of PANI improved thermal stability in this stage.

Although the composites initiate degradation slightly earlier than H_0_, the delayed fourth event indicates a stabilization effect from PANI within the PAM/CMC network. Among them, H_6_ exhibited the highest mass loss in the third stage (25.4%), reflecting stronger contribution of PANI decomposition. Overall, the PAM/CMC/PANI hydrogels exhibited favorable thermal behavior under the conditions investigated, supporting their potential for electroactive applications.

## 4 Conclusions

Semi-IPN PAM/CMC/PANI hydrogels were successfully synthesized, and the APS/ANI ratio was identified as an important parameter governing their swelling behavior, electrical conductivity, and thermal properties. An intermediate APS/ANI condition resulted in the highest electrical conductivity, reaching values on the order of 10^–2^ S cm^–1^ for H_6_, which also exhibited comparatively homogeneous PANI distribution and higher crystallinity than H_5_ and H_7_ matrices. The combined swelling, electrical, spectroscopic, morphological, and structural results demonstrate that the APS/ANI ratio strongly influences the structure–property relationships of PAM/CMC/PANI hydrogels. These findings indicate potential relevance to electroactive and energy-storage-related materials. However, long-term electrical stability, cycling durability, and swelling–drying stability were not evaluated in the present study and should be addressed in future work.
